# Curviness is a better predictor of a woman’s body attractiveness than the waist-to-hip ratio

**DOI:** 10.1038/s41598-024-74265-z

**Published:** 2024-10-04

**Authors:** Ronald Hübner, Emily Sophie Ufken

**Affiliations:** Department of Psychology, University of Konstanz, D-78464 Konstanz, Germany

**Keywords:** Waist-to-hip ratio, Curviness, Curvature, Physical attractiveness, Psychology, Human behaviour

## Abstract

The waist-to-hip ratio (WHR) is commonly used as an indicator of mid-body fat distribution and is often used to answer health-related questions. It has also been suggested that a woman’s WHR can signal her reproductive fitness. This notion is supported by evidence indicating a relation between WHR and a woman’s physical attractiveness. However, it was also acknowledged that the actual fitness cue is the curviness of a woman’s body. While curviness is easy to perceive, it is difficult to quantify. Therefore, the WHR is often considered as a simple measure of body curviness. However, the WHR and curviness are not uniquely related. After replicating results of a pioneering study in this area, we therefore tested whether the WHR or curviness better predicts a woman’s physical attractiveness. As stimuli, we used simple line drawings of women’s bodies, differing in their curviness and width. The results demonstrate that curviness is a better predictor, even though we used a relatively simple curvature-based measure of curviness. This outcome indicates that the WHR is a poor measure of a woman’s body curviness and underscores the need for a more accurate measure of curviness when assessing the physical attractiveness of a woman’s body.

## Introduction

When it comes to describing the human body and judging its attractiveness, proportions often play an important role. This has been the case since ancient times and was further developed in the Renaissance by artists such as Leonardo da Vinci and Albrecht Dürer, who attempted to find a formula that would characterize the perfect human body^1^. Later, in the 19th century, Zeising^2^ claimed that various proportions of the human body are based on the golden ratio, which he considered as most “pleasing”. Ratios are still used today, especially in connection with the female body and its attractiveness^3,4^. One reason for this is that they enable an enormous reduction in shape information and are easy to measure. However, this convenience comes at a price, as ratios are usually also a simplification. In this study, we investigated the cost of using a specific ratio, the body’s waist-to-hip ratio (WHR) rather than its shape to predict women’s physical attractiveness.

### WHR and attractiveness

It is obvious that women differ in their physical attractiveness. This is mainly based on the fact that some properties of specific body parts are considered more beautiful than others. But why is this the case? A promising approach for answering this question is offered by the evolutionary theory of human mate selection. Since women differ in their reproductive fitness, i.e., fertility and health, it is advantageous for men to mate with women of high fitness. Unfortunately, fitness-relevant properties are usually not directly observable. However, for some properties it has been proposed that there are visible cues that more or less reflect a corresponding fitness. It is further assumed that evolution has adapted our brains in such a way that we find women physically attractive whose cues signal high fitness^5–7^.

An intensively investigated example in this respect is the distribution of fat in a woman’s body. Fat is not only important for overall health^8–10^, but also for the ability to conceive and deliver a viable child^11^. However, its effects differ depending on the region of the body where it is located. For instance, fat in more upper regions has some negative effects on general health, whereas fat around women’s hips has fewer negative effect and even some positive effects on pregnancy^12^. Thus, a woman’s health and reproductive success depends to some extent on the distribution of fat in the middle part of her body. Unfortunately, characterizing this distribution is not an easy task for researchers who want to assess fertility and health risks. Therefore, it was a major advancement when Hartz, et al.^13^ published a study showing that the WHR is a useful measure of body fat distribution.

About a decade later, the WHR was then taken up by the evolutionary psychologist Devendra Singh and linked to the physical attractiveness of female bodies. In a highly acclaimed series of studies^14–16^ he used simple line drawings of women’s bodies varying in WHR from 0.7 to 1 in each of three body-weight categories (underweight, normal weight, and overweight) as stimuli and had participants to assess their attractiveness. Singh always found that in each weight category the body with a WHR of 0.7 was the most attractive. Tassinary and Hansen^17^, however, criticized Singh’s studies, because in the stimulus set weight is confounded with hip size, and WHR with waist size. Moreover, there was no body with a WHR below 0.7. In their own study, they therefore used an optimized stimulus set and found, different from Singh, no strong relation between WHR and attractiveness. A later study by Streeter and McBurney^18^, though, could not confirm the results of Tassinary and Hansen^17^.

If the WHR is a fitness cue resulting from evolution, then female bodies with a WHR of 0.7 should be considered most attractive in all cultures. This, however, is only the case to a limited extent. There is some variation across cultures. Chinese men, for instance, seem to prefer female figures with an WHR of 0.6^19^. Moreover, in a recent study, Iranian and Norwegian men were shown to prefer higher WHRs in women compared to Polish and Russian men^20^.

Nevertheless, despite these cultural modulations, there is abundant behavioral evidence in favor of a systematic relation between WHR, health, and physical attractiveness^21,22^. In addition to behavioral studies, electrophysiological studies were also conducted in which event-related potentials (ERPs) were recorded in response to female bodies with different WHRs. In one of the studies, it was shown that female bodies with a WHR of 0.7 triggered greater positivity at the P1 level in male observers than other WHRs, indicating early visual WHR processing^23^. However, the results of this study could only be partially replicated in a later study^24^.

Taken together, many results suggest that the WHR is a simple index of a woman’s reproductive fitness and other characteristics^25^. However, it is difficult to imagine that the WHR is also the psychologically effective criterion for attractiveness. Rather, because the fat distribution largely determines the shape of the mid-body region, it seems more reasonable to assume that its characteristics serves in some way as fitness cue. Indeed, a woman’s “curves” are often seen as the crucial factor for her physical attractiveness^12^. Unfortunately, although curves can be easily perceived, they are difficult to quantify. Therefore, the WHR is usually considered a valid measure of a woman’ s curves in the mid-body region and is used instead. Bainbridge^26^, for instance, in his book “*Curvology: The origins and power of female body shape*”, considered the WHR and its effects in detail, but never analyzed curves. The same applies to other authors who speak of “curviness” or “curvaceousness”^27–29^. This superficial treatment of body shape is probably due to the assumption that it is adequately represented by the WHR, and to the general popularity of ratios.

### Curviness

Of the various terms referring to the properties of curves, such as *curvature*, *curviness*, or *curvaceousness*, we will use *curvature* to refer to the formally defined curvature along a curve, and the term *curviness* to denote the perceived overall degree of curvature.

If we consider the outline of a human body, then the locations used for calculating the WHR are usually points of high curvature. For object perception, such points are most important. Simply connecting them with straight lines makes many objects already recognizable^30^. However, this does not imply that such a reduced information is also sufficient for attractiveness perception. We assume that the entire contour in the area from the chest to the thighs is important for attractiveness. In frontal view, the outline of the body in this region is usually S-shaped (or reversed S-shaped). Such lines are generally perceived as beautiful, as the British artist and theorist William Hogarth (1697–1764) already noted. From a series of S-shaped lines whose curviness increased monotonously, he even declared one line (Number 4) with a specific medium curviness as the “Line of Beauty”^31^, an assertion that has recently been confirmed empirically^32^. For our objective, it is noteworthy that Hogarth also related his Line of Beauty to the attractiveness of the female body. However, he was referring more to the back profile, which also has an S-shaped outline^33^. In a recent study, it was indeed shown that the back profile of a physically attractive woman is highly similar to Hogarth’s Line of Beauty^34^.

In regard to these considerations, it might be more appropriate to directly take curviness as fitness cue reflecting the distribution of body fat. The curviness is not only easily perceptible, but also independent of the body width, in contrast to the WHR. The latter fact allows to consider body width as an independent cue for assessing fitness. Indeed, body width not only indicates the amount of fat around the hips, but also the pelvic width, which is important for successful birthing^35^. Accordingly, Tassinary and Hansen^17^ could show that hip and waist sizes have an independent effect on attractiveness and perceived fertility, which can even be greater than that of the WHR. Recently, the idea of an independent effect of the two WHR components found further support in a study by Lassek and Gaulin^36^.

### Non-uniqueness problem

A serious problem with using the WHR is that bodies of different weight or width can have the same WHR, i.e., the relation between WHR and body shape is non-unique. This is why functional relations between the WHR and attractiveness can only be stated within a given weight or width category. Most important for our objective, however, is that the relationship between the WHR and curviness is also non-unique, because curviness, in contrast to the WHR, is independent of body width, which implies that the WHR cannot be a generally valid measure of curviness. The latter non-uniqueness is somewhat obscured in Singh’s stimuli. To see why this is the case, we took a closer look at how Singh varied the WHR.

**Fig. 1:**
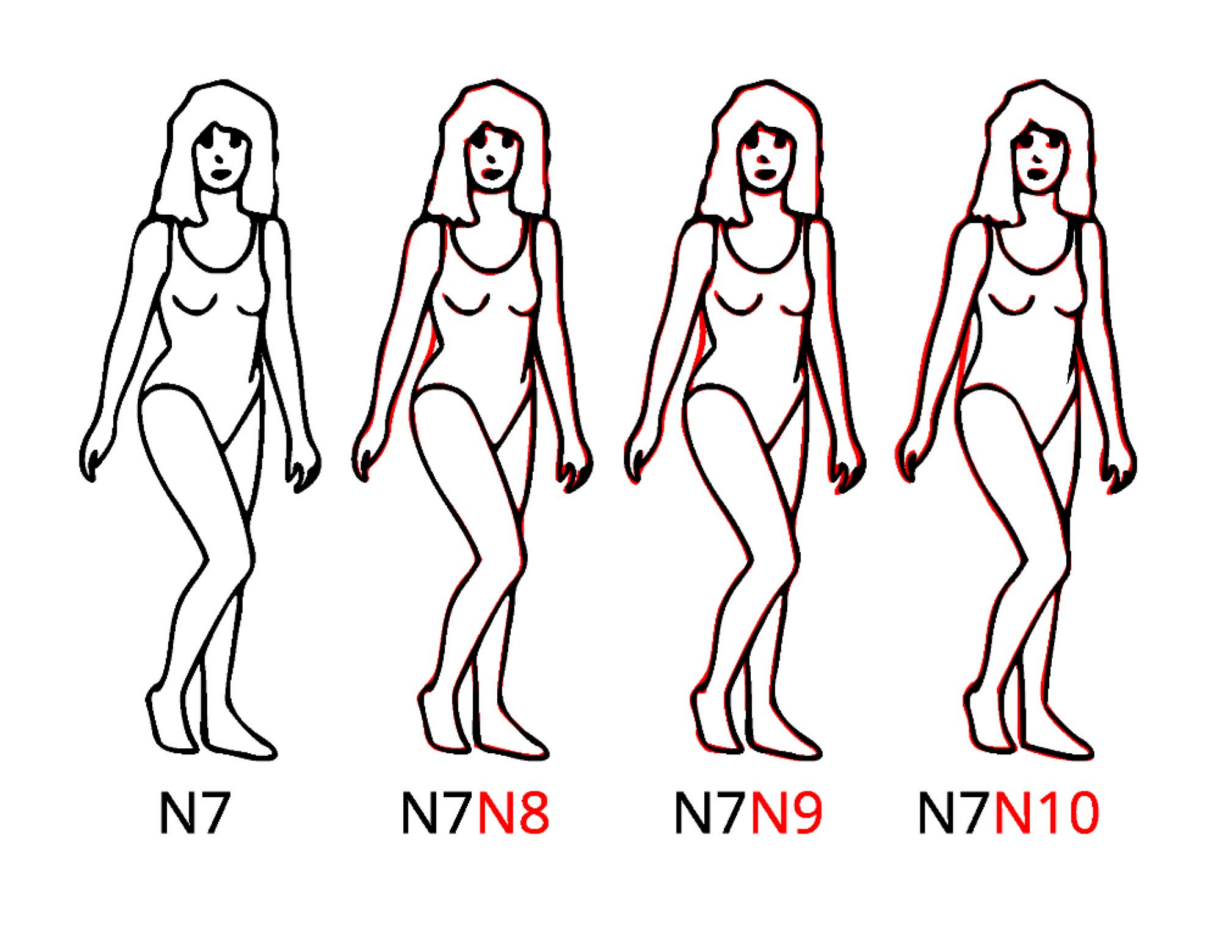
Traced line drawings of female bodies from Singh^15^. The bodies are from the normal weight category (N). Bodies from the WHR category 8, 9, and 10 are superposed with body N7, respectively, to demonstrate how the WHR was varied (see text for details).

On the left side of Fig. 1, one can see a traced version of Singh’s line drawing of a body in the normal-weight category (N) with a WHR of 0.7. First of all, this and the other bodies are special, as they are shown in a certain pose so that only one side of the body is curved. By superimposing body N7 on the bodies with WHR 0.8 (N8), 0.9 (N9), and 1.0 (N10), respectively, as shown in Fig. 1, it becomes clear that the curviness of the waist area on the right side of the body was decreased to increase the WHR. Accordingly, curviness and WHR are strongly correlated within a weight category.

The more critical part, however, is to vary the WHR between body weight categories. In Singh’s and many subsequent studies, body weight was simply varied by modulating body width. If the width of a body is increased, then the WHR also increases. However, by also increasing curviness it is possible to maintain the original WHR. This is the reason why bodies of different widths can have the same WHR. Thus, we have the peculiar situation that within a weight category curviness and WHR vary together, but across weight categories curviness usually varies between bodies with the same WHR. As a consequence, the overall correlation between curviness with WHR is reduced. Consequently, as already mentioned, the WHR cannot be a valid measure of curviness. However, the low correlation between WHR and curviness also offers the possibility to test whether the one or the other better predicts a woman’s physical attractiveness.

**Fig. 2:**
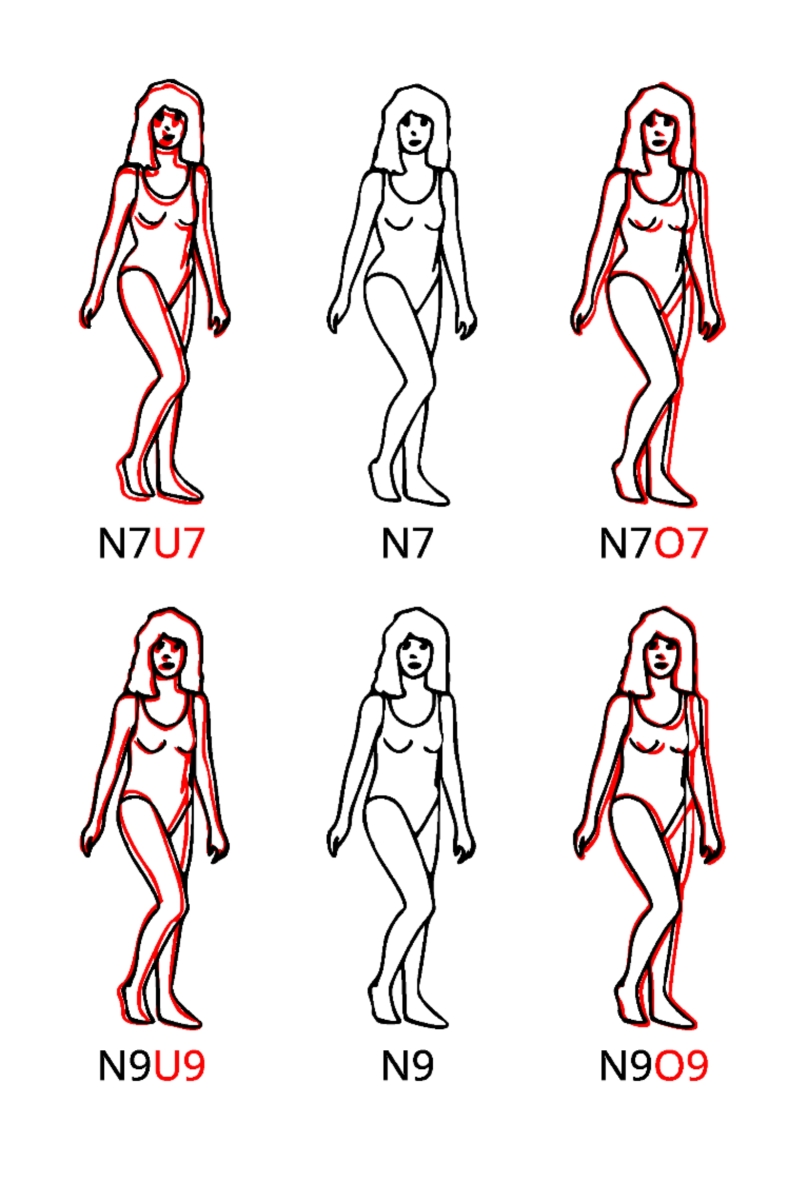
Examples of Singh’s line drawings. In the center column are the normal weight stimuli with a WHR of 0.7 (N7) and 0.9 (N9), respectively. Left and right are the corresponding underweight and overweight stimuli in red. For comparison, the stimuli are drawn on top of N7 and N9 bodies, respectively (U: underweight, N: normal weight, O: overweight).

Given these relationships, it is curious that in Singh’s stimuli the curviness hardly seems to differ between the weight categories. This is demonstrated in Fig. 2, which shows the bodies with a WHR of 0.7 and 0.9, respectively. By superimposing the normal-weight bodies with the corresponding bodies in the underweight (U) and overweight (O) categories, it becomes obvious to what extent the width of the U-bodies is reduced and that of the O-bodies increased compared to the N-bodies. Most importantly, however, is that the curviness of the critical right body part is practically identical across weight categories for a given WHR. How can this be, in view of the relationships mentioned above? To answer this question, we analyzed Singh’s stimuli in detail. We measured the width of the hips and waist and the distance between the maximum and minimum (MMD) extension of the body on the left and right sides in the critical region. The measurements resulted in a WHR of 0.70, 0.70 and 0.79 for the U7, N7 and O7 bodies respectively. The WHR values provided by Singh therefore reflect categories rather than precise individual measures.

To maintain a WHR of 0.7 from N to U, the MMD on the right body side was decreased slightly from 1.52 to 1.35 units, while the MMDs on the left side are identical (0.37, 0.37 units). For obtaining body O7, the MMD on the right body side was slightly increased to 1.55 units, while it decreased to 0.23 units on the left side. The relations between the measures are similar for the WHR 9 category. The WHR is 0.82, 0.82, and 0.86 for the bodies U9, N9, and O9, respectively. The corresponding MMDs for the right side of the body are 0.95, 0.88, and 0.95 units and for the left side 0.30, 0.28, and 0.13 units. Thus, it seems that the bodies were modified within each WHR category in such a way that the curviness of the right body side, which is probably in the focus when evaluating attractiveness^37^, remains rather similar. These analyses demonstrate that in studies that applied Singh’s stimuli, WHR and curviness were almost perfectly correlated, i.e., confounded. This could have encouraged researchers to consider the WHR as a valid measure of curviness.

### The present study

Our considerations show that the WHR and curviness are each relatively simple on their own. The WHR is the ratio of the waist and hip dimensions, while curviness represents the shape of the mid-body outline. However, the relationship between the two characteristics is relatively complex, mainly because the WHR depends on body width, which is not the case for curviness. Because of this difference it does not appear justified to regard the WHR as a valid measure of curviness. Therefore, the question we wanted to investigate in this study was which of the two properties is a better predictor of women’s physical attractiveness. As we have seen, Singh’s figures are not suited as stimuli for this objective, because WHR and curviness are almost perfectly correlated. As a consequence, any relation with attractiveness can be attributed in the same way to either variable, WHR or curviness. Accordingly, we varied curviness and body width independently, which reduced the correlation between WHR and curviness. However, before conducting this critical experiment, we first wanted to replicate Singh’s results with our methods.

In most of Singh’s empirical studies, as well as those using his figures, all 12 drawings were presented simultaneously although in a randomized order. Participants were then asked to rank these stimuli based on several attributes, including attractiveness. However, the simultaneous presentation of all stimuli might have made the experimental manipulations visible to the raters, which could have influenced their ratings^38^. Moreover, data from rankings are non-parametric, and therefore are limited with respect to the application of powerful statistical methods. Furnham, et al.^39^ therefore replicated Singh’s study using a 7-point bipolar Likert scale (‘very unattractive’ to ‘very attractive’). All participants were given a booklet, with three figures on each page. The results were somewhat different from Singh’s. For example, in the overweight category, the body with a WHR of 0.8 was rated as most attractive. Furthermore, in the underweight category, there was a non-monotonicity. However, Furnham et al. included male figures in their stimulus set, which might also have affected the ratings. In view of these inconsistencies, we first (Experiment 1) wanted to examine to what extent Singh’s results can be replicated with the methods planned for our critical experiment, i.e., presenting the stimuli individually in a randomized order in an online study and having participants to rate each stimulus in terms of attractiveness on a visual analog scale.

In Singh’s line drawings it can well be seen how arms, face, and other parts of the body change more or less systematically with body width, which potentially can also affect a body’s attractiveness. Here, we wanted to avoid any potential confounding variables. Therefore, in our critical experiment (Experiment 2) we restricted the body figures to those parts that are relevant for the perception of curviness and the WHR, i.e., we presented torsos, which, for simplicity, we will also refer to as bodies. The crucial question was exactly which body region to consider. Researchers have not been specific in this regard so far when talking about curviness. Given that fitness relevant fat is distributed in the waist and hip area, it seems that the middle region of a body is important. That this region is related to attractiveness is also supported by results indicating that men, while making female attractiveness judgements, look preferentially at the hip size and the curved outline of this region^37^. For our torsos we extended this region somewhat and considered the body region from the chest to the thighs to be relevant. We hypothesized that the curviness of this region strongly determines a body’s attractiveness, and that its effect is stronger than that of the WHR.

Our hypothesis was also motivated by results of a recent study on the beauty of vases^40^, in which we dealt with a similar problem. In his book “*Aesthetic measure*”, Birkhoff^41^ attributed the beauty of S-shaped vases to some of their proportions, one of which resembles the WHR. We could show that the beauty of such vases can better be predicted by curviness than by those proportions^40^.

## Experiment 1

Our first experiment was designed to examine to what extent Singh’s results can be replicated using the methods planned to apply in our second experiment, i.e., presenting stimuli individually in randomized order in an online experiment, and having them rated in terms of attractiveness on a visual analog scale.

### Method

#### Participants

Eighty persons (mean age 28.5 years, *SD* = 6.62, 25 males), most of which were students from the University of Konstanz, participated in the experiment, which was part of a larger online study. The results of the other parts will be reported elsewhere. For participation in the study, which overall lasted about 10 min, the participants received a voucher worth 2 €. The experiment was approved by the Institutional Review Board of the University of Konstanz, Germany (IRB 07-2024), and the experiment was conducted in accordance with the Declaration of Helsinki (1964) and its later amendments. Participants were informed of their right to quit the study at any time without reprisal. Their informed consent was obtained by check-marking a box before the actual experiment started.

#### Stimuli

As stimuli served 12 line drawings, which consisted of paths fitted to the drawings used in Singh’s studies. Examples can already be seen in Figs. 1 and 2. All bodies are shown in the Results section. The correlation between hip width and WHR is 0.913, *t*(10), *p* < 0.001. On the screen the stimuli were presented with a height of 400 pixel.

#### Procedure

The program for the experiment was written in JavaScript and ran in the browser used by the respective participant. At the beginning, participants were briefly introduced to the topic and procedure of the study. After consent and provision of personal data (e.g., gender, age), a specific instruction was presented, and the corresponding task was requested. The stimuli were presented in black on a white 500 × 500 pixel square on the screen in randomized order. To achieve standardized visual quality of stimuli presentation, participants were informed that they had to use a computer. The program stopped if a mobile device was used. The participants’ task was to rate each of the bodies according to how much they liked it (from “Überhaupt nicht schön” (Not beautiful at all) to “Sehr schön” (Very beautiful), on a visual analog scale internally ranging from 1 to 100. We consider these ratings as attractiveness ratings. Although beauty is not equivalent to attractiveness, in most cases it is very similar, and usually easier for participants to understand.

### Results

**Fig. 3:**
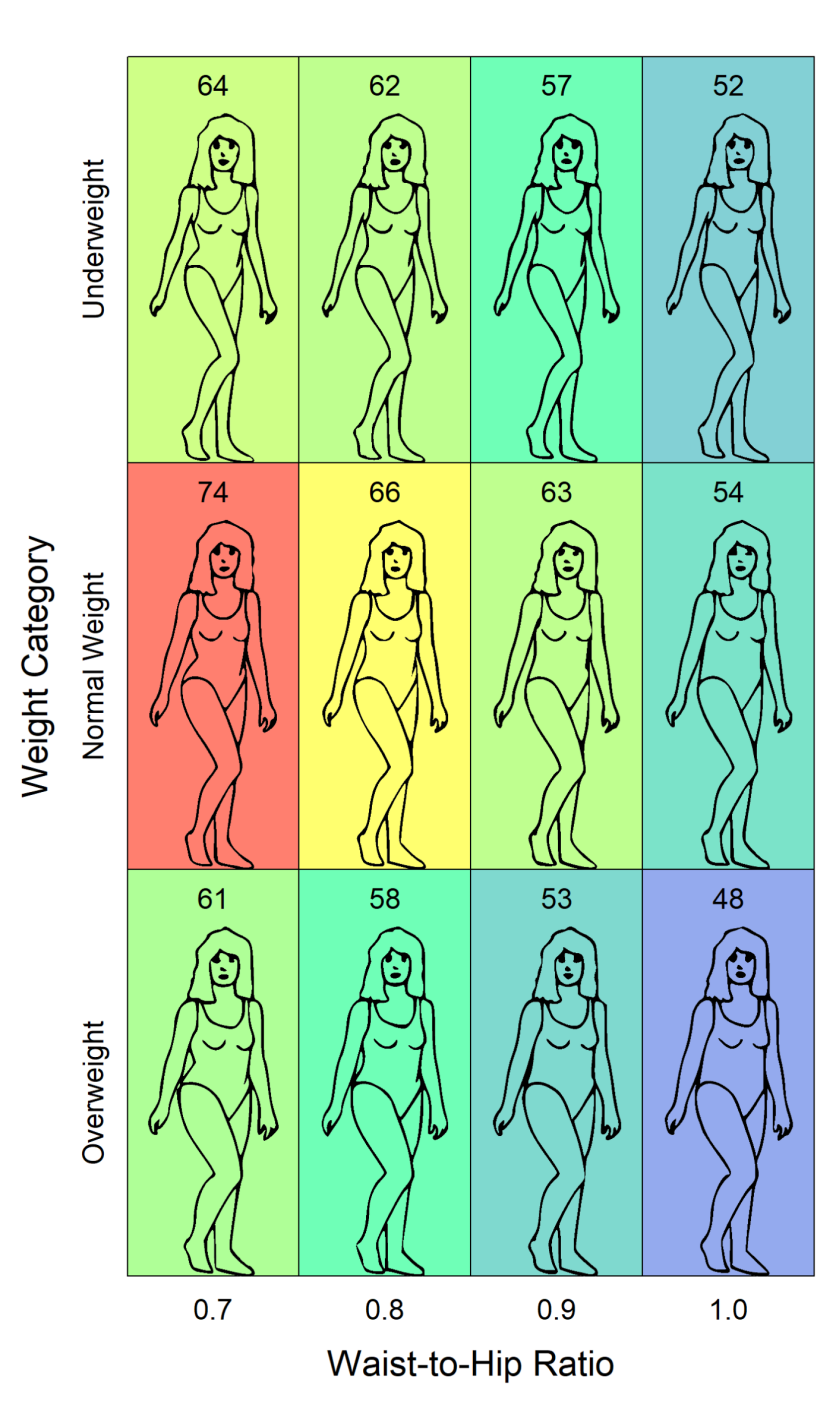
Results of Experiment 1. The average attractiveness ratings are provided as numbers at the top of each panel and are also coded by the background color. The heatmap goes from blue (48) to red (74).

The mean of the attractiveness ratings is 59.3 (*SD* = 22.9). For the individual bodies it ranges from 48 to 74. Figure 3 shows the mean ratings for each body. To evaluate the consistency among the raters, we computed the intraclass correlation (ICC) with the function ‘icc’ from the R package ‘IRR’^42^. As result, we obtained an ICC(C,80) of 0.95 (two-way, average), which indicates a very high rater consistency^43^.

The ratings were subjected to an analysis of variance (ANOVA) with the within-participants variables *WHR* (0.7, 0.8, 0.9, and 1.0) and *weight category* (U, N, and O). It revealed a significant main effect of the variable WHR, *F*(2.06, 163) = 51.6, *p* < 0.001, *GES* = 0.061. The overall mean ratings decreased with an increasing WHR (66.3, 62.0, 57.6, and 51. 3). The main effect of the variable *weight category* was also significant, *F*(1.39, 109) = 11.0, *p* < 0.001, *GES* = 0.029. Normal weight obtained the highest ratings (58.7, 64.2, and 55.0).

However, there was also a significant interaction between the variables, *F*(6.00, 474) = 2.83, *p* = 0.01, *GES* = 0.035. Bonferroni adjusted t-tests revealed that the significance of the rating differences between the WHRs varied depending on the weight category. There was no significant difference between the WHRs 0.7 and 0.8, *t*(79) = 0.928, *p*_*adj*_=1.0, and between the WHRs 0.9 and 1.0, *t*(79) = 2.18, *p*_*adj*_ = 0.192, for the underweight category. For the normal-weight category, there was no significant difference between the WHRs 0.8 and 0.9, *t*(79) = 2.31, *p*_*adj*_=0.138. Finally, for the overweight category, there was no significant difference between the WHRs 0.7 and 0.8, *t*(79) = 2.25, *p*_*adj*_=0.162, and between the WHRs 0.8 and 0.9, *t*(79) = 2.68, *p*_*adj*_ = 0.054.

### Discussion

Despite our different methods, we found similar results as Singh in his studies. Bodies in the normal-weight category were liked more than those in the underweight and overweight categories. Most importantly, in each weight category the body with a WHR of 0.7 was liked most, although the difference from WHR 0.8 was not significant in the underweight and overweight categories. Thus, despite our very different method, we successfully replicated Singh’s results. This also shows that it is irrelevant for the assessment of attractiveness of these stimuli whether all stimuli are present at the same time or not. However, because the WHR category and curviness are largely confounded in Singh’s stimulus set, as demonstrated in the Introduction, the stimuli do not allow to answer the question of which of the two variables is better at predicting a woman’s physical attractiveness. In Experiment 2, we therefore used a different stimulus set to find an answer to this question.

## Experiment 2

**Fig. 4:**
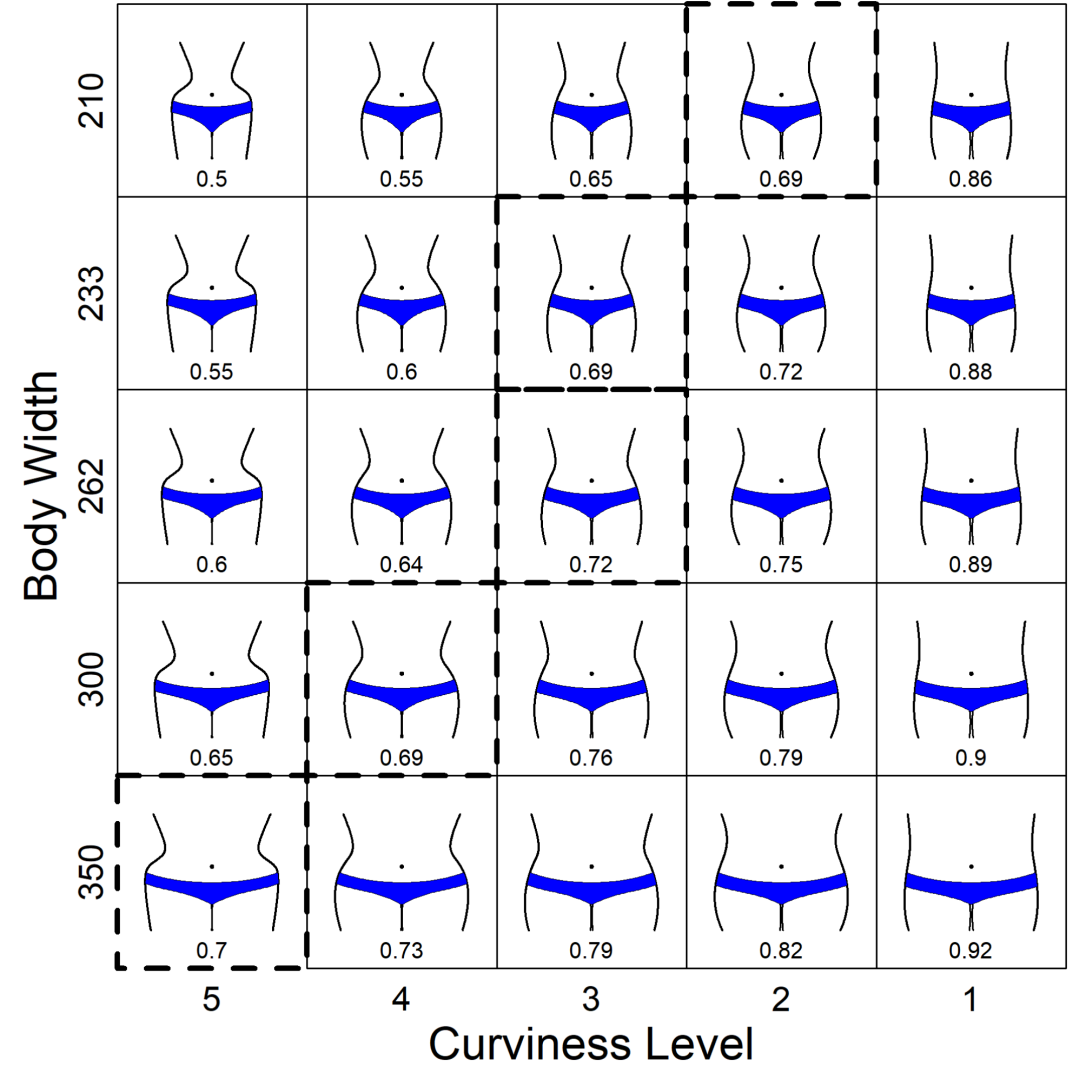
The 25 body stimuli used in Experiment 2, with the number at the bottom indicating the respective WHR. Bodies with a dashed frame have a WHR that is closest to 0.7 for the corresponding width.

In our second experiment we systematically varied, in addition to body width, the curviness of the lateral contours. This had the effect that, in contrast to Singh’s stimuli, curviness and WHR were not perfectly correlated. Finally, we restricted the body stimuli to those parts that are relevant for the perception of curviness and WHR. Our stimulus set is shown in Fig. 4.

According to Singh^14,15^, bodies with a WHR close to 0.7 should be most attractive for a given body width. In Fig. 4, the corresponding bodies are framed by dashed lines. As can be seen, they largely vary in curviness, which is different from Singh’s stimulus set. In contrast, if curviness is the relevant property, as we hypothesized, then the bodies with a certain curviness should be most attractive irrespective of its width. That is, in each row of Fig. 4, the most attractive body should be located in the same column.

### Method

#### Participants

Ninety-eight persons (mean age 28.2 years, *SD* = 8.46, 33 males), most of which were students from the University of Konstanz, participated in the study, which was conducted as first part of a larger online study. The results of the other parts will be reported elsewhere. For participation, which lasted about 10 min, the participants received a voucher worth 2 €. The ethical conditions were the same as in Experiment 1.

#### Stimuli and procedure

**Fig. 5:**
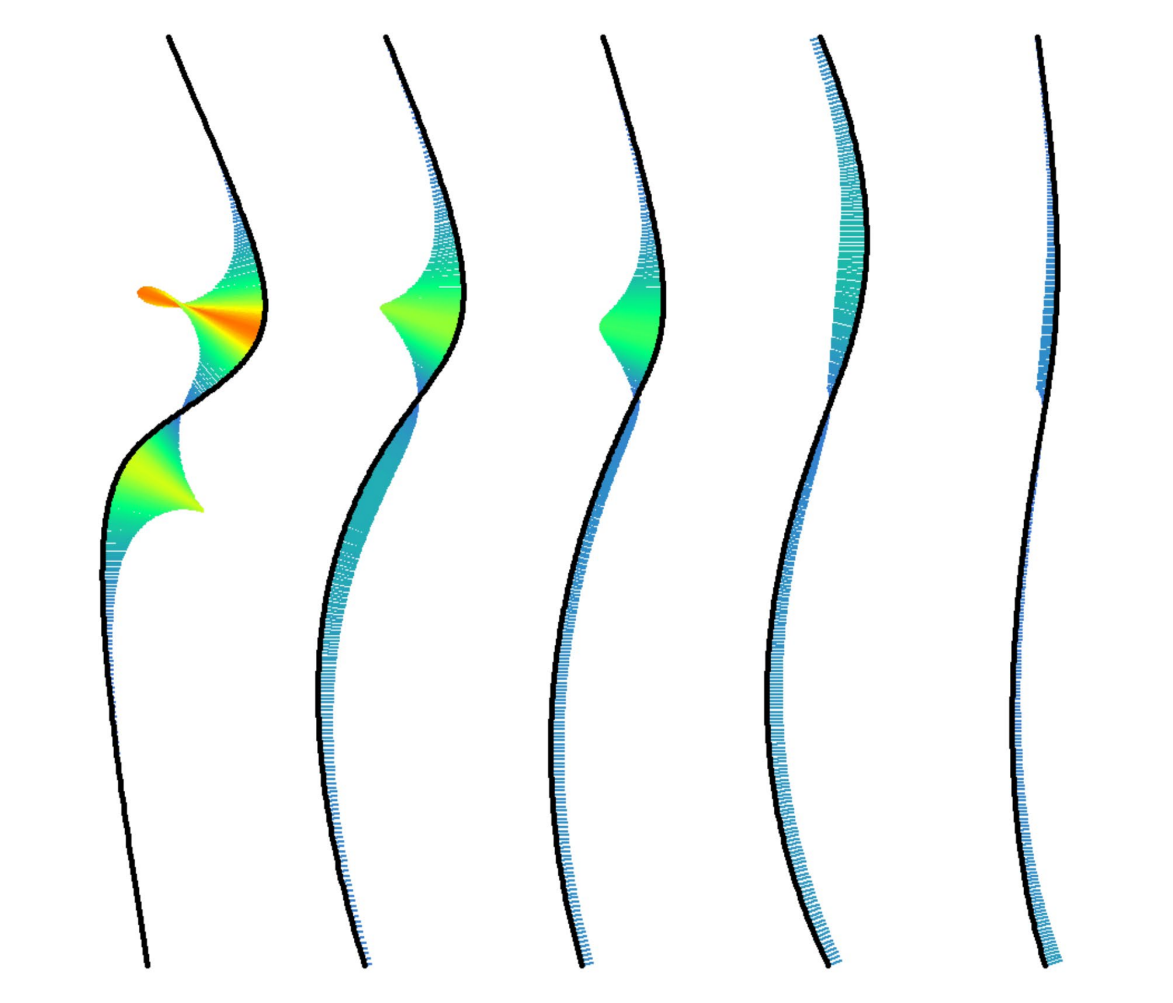
The five left-sided (viewer perspective) body contour lines and their curvature ‘comb’^46^. The curvature along the lines is indicated not only by the (scaled) length of the comb teeth, but also by their color according to a heat map.

Five lines differing in curviness (see Fig. 5) and their respective mirror image were used to construct our stimuli. The lines were obtained by fitting Beziér curves to the left side of corresponding real women’s bodies that we found depicted in photos (frontal view) on the Internet. The use of Bézier curves is standard in computer graphics to represent a curve by a small number of parameters^44^. It also makes it easy to calculate the curvature along the curve (see Fig. 5). The woman with the highest curviness wore a corset similar to that shown on the cover of Nancy Etcoff’s^45^ book “*Survival of the prettiest: The science of beauty”*.

The two-dimensional WHR of the original bodies (ordered by curviness from high to low) was 0.47, 0.53, 0.67, 0.67, and 0.75, respectively. The curviness of the five lines differs in several aspects, not all of which are easy to quantify. To have at least an overall formal measure, we used the R package ‘knotR’^47^ to calculated the mean absolute curvature, simply called *curvature*, of each line, which is 0.01211, 0.0076, 0.00569, 0.00493 and 0.00257, respectively. The mean absolute curvature of each curve corresponds to the average teeth length of its curvature comb (see Fig. 5).

The height of the bodies was 300 pixels. By combining each of the five curviness levels with five body widths (210, 233, 262, 300 and 350 pixels) the 25 stimuli shown in Fig. 4 were constructed. The five width values represent the respective width of the hips and at the same time indicate the maximum horizontal extension of the body. The widths were chosen so that the WHR for the bodies with the highest curviness level varies between 0.5 and 0.7 in steps of 0.05 (see the stimuli in the far-left column in Fig. 4). In our stimulus set body width and WHR are correlated with *r* = 0.430, *t*(23) = 2.29, *p* < 0.05. In contrast, because body width and curvature are independent, they are uncorrelated. However, curvature and WHR are still correlated with − 0.819, *t*(23) = − 6.85, *p* < 0.001.

The procedure was the same as in Experiment 1.

### Results

The mean of the attractiveness ratings across bodies is 43.2 (*SD* = 27.9), where the mean ratings for the individual bodies range from 17 to 69. The consistency among the raters was again very high, ICC(C, 98) = 0.99.

**Fig. 6:**
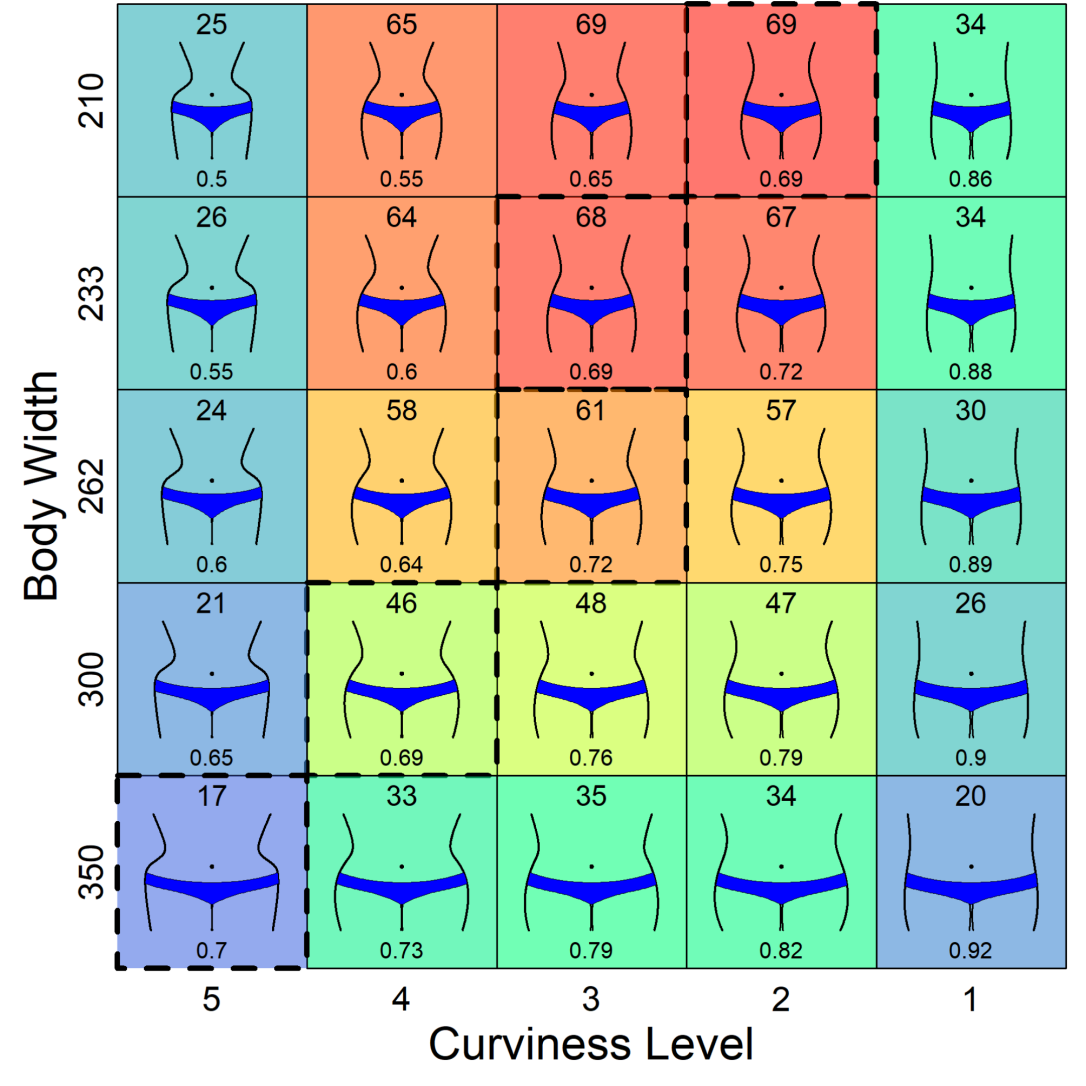
Results of Experiment 2. The mean attractiveness ratings are provided as numbers at the top of each panel and are also coded by the background color. The heatmap goes from blue (17) to red (69). The number at the bottom of each panel indicates the corresponding WHR.

As can be seen in Fig. 6, the maximum-attractiveness prediction of Singh’s WHR hypothesis holds for the three smallest body widths, but increasingly deviates for bodies with a greater width. The deviation is extreme for the largest width, where the body with a WHR of 0.7 not only received the lowest ratings among the bodies of this width, but also among all bodies. In contrast, the prediction of the curviness hypothesis almost fully agrees with the results. The highest mean rating for the bodies in each row is in column three (but note that for the smallest body width, the body in column two has the same mean attractiveness).

However, the hypotheses should not only predict the most attractive bodies for a given width, but also the entire pattern of attractiveness. Therefore, we further analyzed the data by means of linear regression models. Presumably due to the high interrater consistency, linear mixed models led to the same predictions as corresponding simple linear regression models. We therefore only report the results of the latter models. For examining the WHR hypothesis, we used the WHR and body width as predictor variables. In order to take the obvious non-linear relationship between WHR and attractiveness into account^18^, the WHR was entered into the regression equation as a quadratic polynomial. The curviness hypothesis was examined by simply taking the mean absolute curvature (C) of each line to represent its curviness. Since mean curvature, like the WHR, is non-linearly related to beauty^32^, it was also represented by a quadratic polynomial. The polynomial and the width were then used as predictor variables.

In a first step we computed with the R program ‘lm’^48^ the predictive power of the individual variables with respect to attractiveness (A). As a results, width (W) alone (A ~ W) accounts for 27% of the variance in the attractiveness ratings, *F*(1,23) = 8.58, *p* = 0.008. The quadratic polynomial of the WHR (A ~ poly(WHR,2)) accounts for 28% of the variance, *F*(2,22) = 4.26, *p* = 0.027, while the quadratic polynomial of curvature (A ~ poly(C,2)) accounts for 65% of the variance, *F*(2,22) = 20.3, *p* < 0.001. Thus, of the three variables, curvature explains by far the largest part of the variance in attractiveness.

**Fig. 7:**
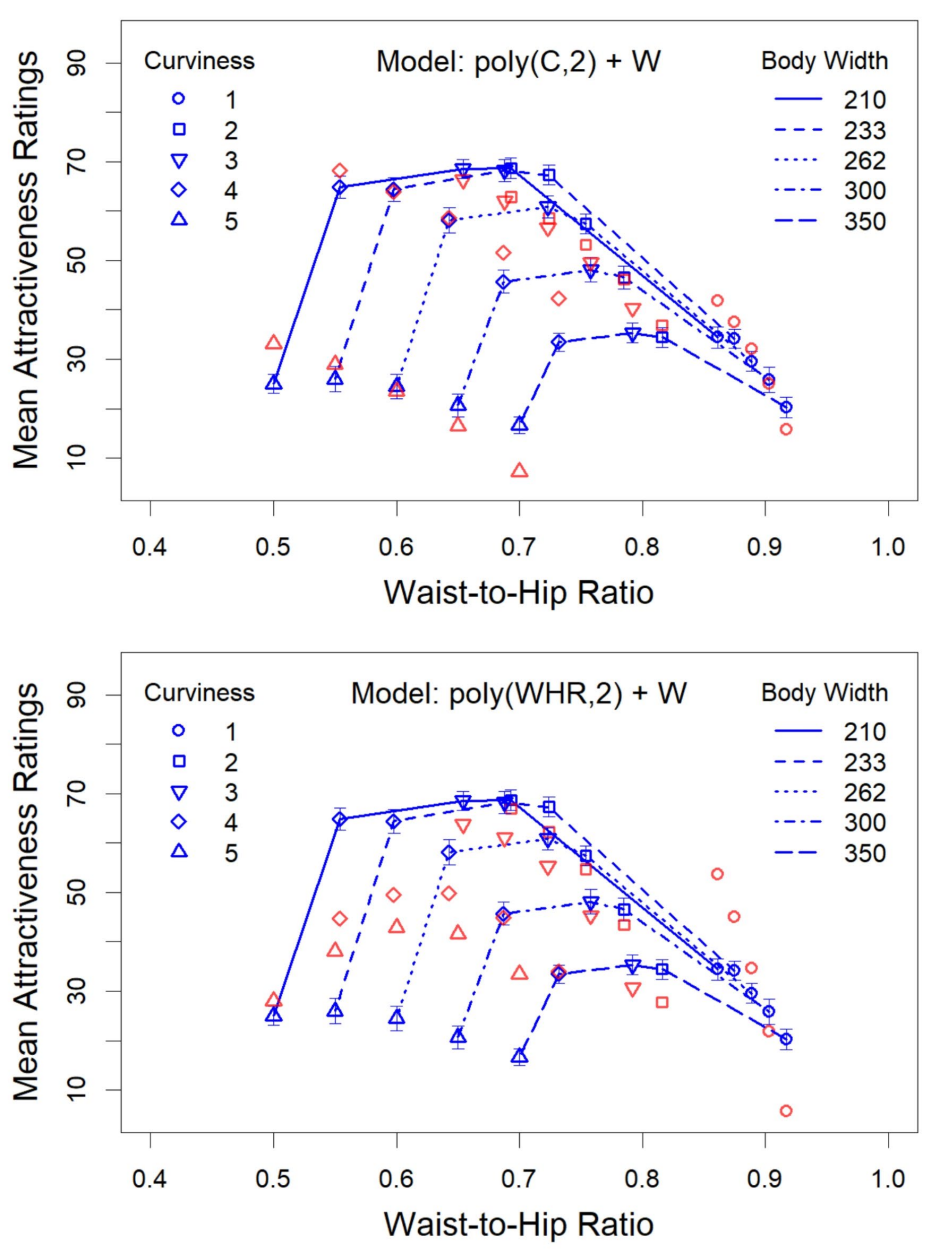
The graphs in the two panels show the mean attractiveness ratings for each body width as a function of the WHR. The red symbols in the upper and lower panel represent the predictions by the WHR and the curviness model, respectively. For more details see the text.

In a second step we examined to what extent the predictions improve by combining the variables. When we used the WHR and width as predictor variables (A ~ poly(WHR,2) + W), then the prediction largely improved, *F*(3, 21) = 12.0, *p* < 0.001, *R*^2^ = 0.631. In Table 1, the coefficients of the model and measures of their reliability are shown. As can be seen, the quadratic component of the WHR is significant, while the linear one is not. The linear component is nevertheless important. If we exclude the linear component, *R*^2^ drops from 0.631 to 0.278. The reason presumably is that WHR and width are strongly correlated, so that the linear WHR component of the polynomial serves as suppressor variable. The details of how the WHR model performs can be assessed by inspecting the upper panel of Fig. 7. In the panels in this figure the WHR is used for the x-axis, which differently stretches the graphs for each body width. The higher the curviness level of a body, the more the graph is stretched towards lower WHRs. According to the WHR hypothesis, the maximum of each graph should lie at a WHR of 0.7, which is obviously not the case for all curves. In addition, most of the other predictions are also far off the data points.

**Table 1:** Summary of regression model for the WHR hypothesis (A ~ poly(WHR, 2) + W).

Coefficient	Estimate	Std. Error	t-value	*p*
Intercept	109	14.8	7.33	< 0.001***
WHR	3.74	13.0	0.286	= 0.777
WHR^2^	−54.6	12.1	−4.52	< 0.001***
W	−0.241	0.054	−4.47	< 0.001***

***p < 0.001.

For modelling the data according to the curviness hypothesis, we used a quadratic polynomial for curvature and the body width as predictor variables. The corresponding regression model (A ~ poly(C,2) + W) explains a large part of the variance, *R*^2^ = 0.920, *F*(3, 21) = 81.0, *p* < 0.001. As can be seen in Table 2, all components of the polynomial are significant. The Akaike Information Criterion (AIC)^49^ is 161, which is also much lower than the AIC of the WHR model, which is 199. The performance of the curviness model can be seen in the lower panel of Fig. 7. Obviously, its performance is much better, compared to that of the WHR model, which is also confirmed statistically by testing the significance of the improvement by adding the predictions of the curviness model to the WHR regression model^50^, *F*(20, 1) = 104, *p* < 0.001.

**Table 2:** Summary of regression model for the curviness hypothesis (A ~ poly(C,2) + W).

Coefficient	Estimate	Std. Error	t-value	*p*
Intercept	93.4	6.03	15.5	< 0.001***
C	−24.0	5.43	−4.42	< 0.001***
C^2^	−66.9	5.43	−12.3	< 0.001***
W	−0.185	0.022	−8.47	< 0.001***

***p < 0.001.

It is worth mentioning that, if we also include interaction terms into the regression model of the WHR hypothesis (A ~ poly(WHR,2)*W), then *R*^2^ increases to 0.879, and the AIC decreases to 175. However, this model not only has huge collinearities due to the correlation of body width with the other variables, but it also loses its characteristic that the highest attractiveness is predicted for bodies with a WHR of 0.7. If we add interaction terms to the curviness model, then *R*^2^ increases to 0.971, and the AIC decreases to 140. Thus, although the difference between the predictions of the two hypotheses decreases, it still remains considerably. Because increasing the complexity of the models also leads to overfitting, we think that the additive models represent the hypotheses more adequately.

### Discussion

The results show that the WHR hypothesis has some predictive values for bodies with a smaller width but fails for those with a larger width. This speaks against the idea that within a weight category always those with a WHR of 0.7 are preferred. Rather, our results indicate that a woman’s body, irrespective of its weight, is liked most if it has a specific curviness. Although the advantage of the curviness hypothesis in this respect, i.e., in predicting the most attractive body within each weight category, is not very strong, it becomes more obvious when it comes to predicting the attractiveness of a large variation of body shape.

In Fig. 7, where the mean attractiveness ratings are plotted as function of the WHR in dependence of body width and curviness level, the inverted U-shaped relation between curviness and attractiveness can clearly be seen, and that this relation systematically changes with body width. The inverted U-shape is stretched upwards and to the left with a decreasing curviness. It can also be seen that, different from the assumption of the WHR hypothesis, the bodies with a WHR of 0.7 are not generally preferred for each body width. Especially for the broader bodies the WHR of the most preferred bodies deviates considerably. The poor explanation of the data by the WHR hypothesis becomes even clearer when we look at all predictions (red symbols) of the corresponding regression model (see top panel in Fig. 7). The deviations of the predicted values from the empirical ones are substantial. This is especially the case for the smallest and the two largest curviness levels, which is different for the curviness model, where we have a good fit and at the same time curviness level 3 always has the highest ratings for each width. These predictions far exceed those of the WHR hypothesis, which speaks strongly in favor of the curviness hypothesis.

## General discussion

After the WHR was introduced as a measure of mid-body fat distribution and applied to investigate various aspects of physical health^13^, Singh^14,15^ proposed that women with a WHR of 0.7 are the most attractive within a given body-weight category. Based on evolutionary ideas, he explained this relationship by assuming that the WHR is a reproductive fitness cue indicating fertility and health, and that men who chose mates on the basis of such cues out-reproduced men who did not. However, it was also more or less tacitly admitted that the curviness of a female’s body is probably the actual fitness cue. Unfortunately, although easy to perceive, curviness is difficult to quantify. Therefore, the WHR was used as a simple measure to represent the curviness of a body.

That the WHR can be used as a valid measure of curviness is also suggested by Singh’s stimuli, because the body figures within the same WHR category have an almost identical curviness. In general, however, this correspondence does not hold^17^. The problem is that the relation between WHR and curviness is non-unique, i.e., the same WHR can occur for bodies with the same curviness but of different weight, or, in case of line drawings, of different width. Or to put it another way, because maintaining a certain WHR with an increasing body width is only possible by increasing the body’s curviness, the correlation between curviness and WHR is limited. Accordingly, the WHR cannot be a valid measure of curviness. However, the limited correlation between WHR and curviness offers the possibility to test which of the two better predicts a woman’s physical attractiveness. Such a test was the main objective of the present study. As Singh’s stimuli are not suited for answering this question, we used a different set of drawings in which curviness and WHR are less correlated.

However, because the results obtained with Singh’s stimuli, depended partly on the applied method^39^, we first wanted to test to what extent they can be replicated with our methods. In Experiment 1, we therefore used Singh’s stimuli and had participants to rate the bodies, presented in random order, in terms of attractiveness in an online-experiment. As a result, the mean ratings are surprisingly similar to those observed in Singh’s studies, indicating that the considered methodological differences between the studies have little effect. In each weight category mean attractiveness increased with a decreasing WHR. Consequently, in each category the body with a WHR of 0.7 was rated as most attractive, although the difference to the body with a WHR of 0.8 was not always significant. Thus, by and large our results confirm Singh’s WHR hypothesis. Nevertheless, because WHR and curviness are highly confounded in Singh’s stimuli, it remained open which of the two properties was responsible for that outcome.

In Experiment 2, we therefore investigated whether the WHR or the curviness of the female body is more important for a woman’s physical attractiveness. As stimuli, we used simple line drawings of female bodies (torsos) that varied in width and curviness in five stages, respectively. According to the WHR hypothesis, bodies with a WHR of 0.7 should be the most attractive. This, however, was not generally the case in our data. Rather, as predicted by the curviness hypothesis, bodies with a certain curviness were rated as most attractive.

A useful hypothesis, however, should not only predict the maxima of attractiveness, but also the attractiveness of any female body. In this respect the curviness hypothesis was also superior. Results of linear regression analyses revealed that curviness, represented as a quadratic polynomial of the mean absolute curvature, along with body width, explained 92% of the variance in the attractiveness ratings. In contrast, the quadratic polynomial of the WHR, along with body width, explained only 63%.

The importance of curviness is especially evident when considering extreme cases. For example, the difference in curviness is largest between levels 4 and 5 (see Fig. 4), which corresponds to a large difference in the attractiveness ratings. This demonstrates that curviness affects attractiveness substantially regardless of body width. In contrast, there is only a small difference in WHR between the corresponding body pairs for each width. This explains the large prediction errors of the WHR hypothesis for these data (see upper panel in Fig. 7).

Taken together, the findings from Experiment 2 show that the curviness of a female body substantially and systematically influences its attractiveness, with a further modulation effect from the body’s width. These relationships are not only clearer, compared to the WHR, but also have better predictive power for the attractiveness of a female body. This clearly demonstrates the cost of relying on a simple ratio such as the WHR as a measure of curviness.

In view of our results that curviness successfully affects a woman’s attractiveness, we also compared our lines with Hogarth’s seven lines^31^. Computing the root-mean-square deviation revealed that the lines with curviness levels 2 and 3, which correspond to the highest rated bodies, were not similar to Hogarth’s line number 4, i.e., the Line of Beauty. Rather, it is the line with curviness level 4 that is most similar to that line. This demonstrates that, while the back profile of a highly attractive woman resembles the Line of Beauty^34^, lines with less curviness were favored for the side profile of a female’s body in frontal view. It could be speculated that this preference arises from the fact that the lines were viewed in conjunction with their mirrored counterparts. However, in our study on vases^40^, the Line of Beauty was most similar to the outline of the most preferred vase. It therefore remains an open question why the outline of the most beautiful female body was less curved than the Line of Beauty.

## Conclusion

Theoretical considerations suggests that the waist-to-hip Ratio (WHR) cannot serve as a valid indicator of curviness, contrary to what is frequently assumed, either implicitly or explicitly. Consequently, one of these two attributes must be a more effective predictor of a woman’s body attractiveness. The results of this study clearly show that curviness is far superior to the WHR in this respect.

### Limits and future directions

Despite its important findings, our study also has some limitations. Firstly, our stimuli were simple line drawings of female torsos in frontal view, which is appropriate for our objectives, but rather unusual. Most other studies have used line drawings of whole bodies, photographs of real women’s bodies or torsos, or corresponding 3D models. Future studies must therefore show whether the advantage of curviness for predicting attractiveness also generalizes to more ecologically valid stimuli.

Secondly, one of our criticisms of the WHR was that it is merely a single number, a ratio, that is assumed to capture the curviness of a woman’s body. As alternative, we proposed to use curviness directly. However, in our data analyses we also represent the curviness by a single number, the mean absolute curvature of the S-shaped body outline. We admit that this measure can only be a first simple approximation, which is far from satisfactory. However, it was sufficient for our current objectives, and already superior to using the WHR. It should be noted that the WHR represents the curviness of a body shape in pictures only by two points along each curved line. In contrast, our measure, the mean absolute curvature, takes all points along the line into account, thereby providing a summary statistic that includes more information than the WHR. Nevertheless, it is clear that a more sophisticated measure should be developed for representing a body’s curviness.

Thirdly, as mentioned in the Introduction, bodies with a WHR of 0.7 are not most attractive in all cultures. However, the better prediction of attractiveness by curviness compared to the WHR could nevertheless hold true in all cultures. Whether this is the case needs to be shown in future studies.

Fourthly, in addition to the WHR, there are other body characteristics that are related to health and fertility and are therefore considered fitness indicators. In particular, the Body Mass Index (BMI) is intensively discussed as an alternative to WHR^51,52^. We did not discuss this topic as we were only interested in the shape characteristics.

## Data Availability

The data collected in the current study are available in the OSF repository: https://osf.io/5m2fe/?view_only=fd46dec2159d4715ab490de9e75e7213.
